# The Associations between Structural Treatment Characteristics and Post-Treatment Functioning in Compulsory Residential Youth Care

**DOI:** 10.1007/s10566-011-9152-8

**Published:** 2012-02-10

**Authors:** Karin S. Nijhof, Ad A. Vermulst, Jan W. Veerman, Coleta van Dam, Rutger C. M. E. Engels, Ron H. J. Scholte

**Affiliations:** 1 Developmental Psychopathology, Behavioural Science Institute, Radboud University Nijmegen, P.O. Box 9104, 6500 HE Nijmegen, The Netherlands; 2 Developmental Psychopathology, Radboud University Nijmegen, Nijmegen, The Netherlands; 3 Praktikon, Nijmegen, The Netherlands

**Keywords:** Treatment characteristics, Residential youth care, Post-treatment functioning

## Abstract

**Background:**

In 2005 a new compulsory residential treatment program was developed for adolescents in need for protection against themselves or their environment.

**Objective:**

The aim of the present study was to examine the association of structural treatment characteristics of this new residential treatment program (i.e., duration of treatment, discharge status, and group composition in terms of sex) with post-treatment functioning. Additionally, the number of pre-treatment risk factors was included in the model.

**Method:**

A total of 301 adolescents (174 boys, 127 girls), with a mean age at time of admittance of 15.50 (SD = 1.26) participated in this study. The number of risk factors was derived from treatment files of the adolescents at time of entrance. Six months after discharge, adolescents participated in a telephone interview to measure ten post-treatment variables indicating how well they were doing.

**Results:**

The results showed that duration of treatment was related to post-treatment living situation, in that adolescents who were in treatment for shorter durations were more likely to live on their own after treatment. For discharge status, findings suggested that adolescents who were regularly discharged had more frequent contact with their family; however, they also showed higher alcohol consumption 6 months after treatment. Group composition was related to the girls’ official offending, indicating that girls placed in mixed-sex groups showed significantly fewer official police contacts than did girls in girls-only treatment groups.

**Conclusion:**

Overall, structural treatment characteristics were hardly related to the adolescents’ functioning after treatment. Suggestions for future research are discussed.

## Introduction

A small percentage of adolescents in the Netherlands show severe, complex behavior problems. These adolescents are in need of residential care because they must be protected against themselves (e.g., suicidal behavior) or against the environment (e.g., abusive parents, pimps). They often do not accept help and will withdraw themselves from treatment. Prior to 2005, compulsory residential care (i.e., a secure setting) only existed within the juvenile judicial care. Existing residential and psychiatric institutions within the regular youth care were not able to deal with the complexity of problems demonstrated in this specific group. As a result, these adolescents were placed in juvenile detention centers; not because they were convicted for a crime, but to protect them against themselves (e.g., suicidal behavior, automutilation) or their environment (e.g., pimps, abusive parents). Based on a national study investigating this subgroup of adolescents in the juvenile detention centers it was concluded that a new form of compulsory residential care should be developed for two reasons. First, adolescents did not receive the appropriate care for their behavior problems, and second, there was a high risk of imitation of criminal behavior. Therefore, in 2005, a new compulsory residential treatment program was developed for adolescents aged 12–18 years. The difference between the new residential treatment program and existing residential treatment is that the new residential treatment takes place in a secure setting and that restrictions can be imposed (e.g., a restraining order, a time out, temporarily transfer, physical holding). The present study aimed to examine the influence of duration of treatment, discharge status, and group composition (i.e., boys-only, girls-only, mixed-sex) of this new treatment program on post-treatment functioning.

The theoretical description and content of the intervention are described in detail (Van der Poel et al. 2008). In general, the new treatment program exists of a basic treatment including a place to live (treatment group existing of 10–12 adolescents), education and daily activities. The basis treatment program is based on two theoretical models: the social competence model (Slot 1988; Slot and Spanjaard 1999) and the ecological model of Bronfenbrenner (1979, 1994). The social competence model includes that intervention programs should focus on removing the risk factors and introducing protective factors (Masten 1994). The ecological model distinguishes high risks on the individual, family, and peer group levels. This model implies that treatment of adolescents with multiple problems should include a multimodal approach. Next to the basic treatment, a range of additional evidence-based individual interventions (e.g., aggression-regulation training, trauma therapy, medication, cognitive behavioral therapy, substance abuse counseling) as well as family interventions or support (e.g., Functional Family Therapy, Multi System Therapy, Parent Management Training Oregon, parental support) can be offered based on the needs of the adolescents and their families.

The new treatment program is characterized by stages from more to less restrictive care, with the first stage focusing on future orientation. The second stage involves behavioral change and includes encouraging prosocial behaviors and discouraging antisocial behaviors. The third stage focuses on training and preparing for the future and stage four includes the transfer to a new living situation and after care. The main goals of treatment are to give adolescents a safe place to live in society, daily activities (i.e., school engagement or a job), and develop and maintain a stable living situation where adolescents have positive contacts with their family and peer group (Van der Poel et al. 2008).

The adolescents admitted to the new residential treatment program show severe problem behaviors (Nijhof 2011); 98% showed externalizing problems, of which 67% also showed internalizing problems. About 70% have had contact with the police before admittance. Concerning substance abuse, 59% used soft drugs on a regular basis, 17% hard drugs and 18% showed problematic alcohol use. Regarding the families, 36% of the mothers and 17% of the fathers showed severe psychiatric problems. Abuse within the family was 30 and 22% of the adolescents witnessed violence between parents. Concerning the friends, 60% had a deviant peer group. Related to this 30% of the girls was victim of forced prostitution (see for group care reporting guidelines Lee and Barth 2011; Weems 2011).

The duration of the new treatment program is generally 1 year. The underlying assumptions are that adolescents should not be admitted for a longer period than is needed and they should return to a normal environment in the short-term, with counseling and after care available. A review of Frensch and Cameron (2002) found that a shorter duration of treatment is positively related to treatment outcomes. Leichtman et al. (2001) investigated a short-term residential treatment (mean durations between 3 and 4 months) for adolescents with severe problem behaviors and found that these adolescents showed significant improvement, which was maintained 1 year after discharge. Other studies revealed that adolescents, living independently after discharge, experienced the longest duration of treatment (Trout et al. 2010), followed by adolescents who returned to the family, adolescents who went to intermediate settings (e.g., foster care, group homes), and adolescents who were transferred to residential or judicial settings. Adolescents living independently and those living with the family after discharge also showed significantly lower rates of problem behaviors compared to the other two subgroups. Further, compared to the other groups, gang behavior, drugs use, and sexual behaviors occurred more often among adolescents who were sent to restrictive residential settings during the last 8 weeks before discharge.

Concerning discharge status, Trout et al. (2010) found that 55% of the adolescent discharges in residential care were planned and treatment goals were met. This means that 45% of the discharges were unplanned, for example, the adolescents ran away or were transferred to more restrictive settings. Adolescents who went to more restrictive settings (i.e., prison, detention or correctional centers, residential treatment, or drug and alcohol rehabilitation) more often grew up in families with high risk factors (e.g., parental criminality, sexual abuse, family addictions) (see also Stage 1998) and discharge was often unplanned. Scholte and Van der Ploeg (2000) compared adolescents who completed treatment to those who dropped out prematurely and found that 42% of discharges were planned, 7% of adolescents were still admitted at the time of data collection, and 51% of discharges were unplanned. In addition, adolescents discharged successfully were in treatment for a longer duration compared to adolescents who dropped out of treatment prematurely. Of note, Scholte and Van der Ploeg found no significant differences regarding age, sex, externalizing and internalizing problems at time of entry, or family risk factors for adolescents who left treatment according to plan compared to those who left unplanned. Differences were, however, found for the adolescents’ peers, in that the peers of adolescents who left unplanned had higher levels of antisocial behavior than did peers of the adolescents who successfully completed treatment (Scholte and Van der Ploeg 2000).

Despite the finding of Scholte and Van der Ploeg, who found no sex differences, most studies did found important sex differences. Previous studies showed that girls entered residential institutions with more severe problem behavior. Specifically, girls demonstrate higher rates of individual problem behaviors and come from more dysfunctional families (e.g., Connor et al. 2004; Doerfler et al. 2009; Handwerk et al. 2006; Nijhof 2011). Girls are also more likely to have sexually traumatic experiences, such as sexual abuse (e.g., Doerfler et al. 2009). Moreover, due to the more dysfunctional families girls come from, they are also more at risk to develop problematic interaction patterns that can result in more problematic peer relations (Leve and Chamberlain 2005). In addition, girls seem to profit less from residential treatment compared to boys (Frensch and Cameron 2002). These findings suggest that boys and girls admitted to residential treatment might need a different approach (Connor et al. 2004; Doerfler et al. 2009). Surprisingly, as far as we know, previous studies have not included group composition in terms of sex as a predictor of outcome success of residential treatment for adolescents with severe behavioral problems. Few studies investigated the influence of group composition within adult samples. Studies investigating the differences between mixed-sex and single-sex, mostly women-only, reported differences between women in single-sex and mixed-sex treatment groups, in that women in single-sex programs showed more individual problems with drugs and alcohol (Greenfield et al. 2008). Additionally, these women were more often physically abused, experienced shorter duration in treatment, and were more likely to not complete treatment than were women in mixed-sex groups. Moreover, women with substance abuse problems and psychiatric symptoms improved more in single-sex programs than in mixed-sex treatment. Niv and Hser (2007) reported that women in single-sex treatment were also less likely to have been arrested after treatment compared to woman in mixed-sex treatment. Furthermore, according to the women’s own perceptions, they felt safer and more comfortable in single-sex treatment groups (Kauffman et al. 2010) and were more satisfied with the single-sex treatment (Greenfield et al. 2007). It is, however, unclear to what extent group composition is related to outcomes of adolescents in residential care.

The primary aim of the present study was to examine the relationship between structural treatment characteristics including duration of treatment, discharge status, and group composition, and post-treatment functioning. The number of pre-treatment risk factors on the individual, family, and environmental levels was included in this study because these numbers are related to post-treatment functioning (Nijhof et al. 2011). Previous studies found a relation between discharge status and duration of treatment, however, for the new residential treatment program it is unknown to what extent these characteristics are associated with treatment outcomes. In accordance with Trout et al. (2010), who examined a comparable sample, we hypothesized that adolescents with a longer duration of treatment showed more positive outcomes (e.g., more frequent contact with the family, living in the family of independently, less use of drugs, less police contacts). Furthermore, in line with earlier findings on discharge status and treatment outcomes, we expected that adolescents with a regular discharge status would show more positive outcomes. The influence of group composition in terms of girls-only, boys-only and mixed-sex treatment groups on treatment outcomes has rarely been studied. This is surprising since studies examining residential care did find sex differences in problem behavior as well as in treatment improvement. Therefore we included group-composition in our study. Based on findings within adult women treatment groups, we expected better treatment outcomes for girls in single-sex treatment groups than in mixed-sex treatment groups. For boys, we did not expect these differences between single-sex and mixed-sex treatment groups.

## Methods

### Participants

Of all 301 adolescents who participated in the present study, 57.8% were boys. The mean age of participants was 15.50 (SD = 1.26). Of 34.9%, at least one of the parents was born in a non-Western country, for 54.2%, both parents were born in a Western country, and for 11%, the ethnicity was unknown. At time of entry, a mother figure was present (e.g., biological mother, stepmother, foster mother) for 95% of the adolescents, for 4%, no mother figure was present, and for one girl, it was unknown. For 75% of the adolescents, a father figure was present (e.g., biological father, stepfather, foster father), for 23%, no father figure was present, and for 2%, it was unknown whether a father figure was present. Concerning the treatment groups, 24.9% of the adolescents were admitted to girls-only treatment groups, 43.5% to boys-only treatment groups, and 31.6% were admitted to mixed-sex treatment groups.

### Measures

#### Risk Factors

A scoring scheme, based on the Scoring scheme for Demographic Information (Flipse, 2000) was extended to include several questions related to parental or adolescent problem behaviors (Orobio de Castro et al. 2002; Boendermaker et al. 2004), was used to measure adolescent risk factors on different areas before admittance to the new treatment program. Risk factors were measured on three different domains; individual, family, and environmental. The individual domain included risk factors with regard to externalizing and internalizing problems, substance use, life events, and non-adequate sexual behaviors. Risk factors on the family domain concerned structural risk factors (e.g., number of children, relationship between parents), risk factors related to the parenting situation (e.g., quality and stability of the parenting environment), and parental problems (e.g., illnesses, addictions, criminality). Risk factors on the environmental domain referred to being involved with deviant peers and sexual abuse outside the family. All risk factors were registered as present or absent. A total score was calculated for each domain to obtain the number of individual, family, and environmental risk factors.

#### Structural Treatment Characteristics

Three structural treatment characteristics were included in the present study: duration of treatment, discharge status, and the sex composition of treatment groups. Duration of treatment was calculated by subtracting the date of admittance from the discharge date. Discharge status was a dichotomous variable, in which ‘0’ represented a regular discharge and ‘1’ a non-regular discharge. The group compositions included boys-only treatment groups, girls-only treatment groups, and mixed-sex treatment groups.

#### Post-Treatment Functioning

Self-reported structured interviews via telephone were completed approximately 6 months after the adolescent left the residential treatment. Ten indicators were assessed to give an insight into how well the adolescents were doing on several aspects after discharge. These indicators are described below and included living situation, frequency of family contact, quality of family contact, daily activity, social network, the use of soft drugs, alcohol use, internalizing problems, self-reported police contacts, official police contacts.

#### Living Situation

Adolescents were asked whether they were living on their own, in a family situation, or in a residential or judicial setting 6 months after discharge. Based on these three types of living situation two dummy variables were constructed. A distinction was made between residential or family (score 0) versus independent (score 1) living situation and residential or independent (score 0) versus family (score 1) living situation.

#### Intensity of Family Contact

Adolescents were asked how often they had contact with their parents ranging from ‘1’ hardly or none to ‘4’ daily. Higher scores indicated more frequent contact with parents.

#### Quality Family Contact

Adolescents were asked about the quality of the relationships with both their mother and father. The ratings varied between ‘0’ not a good relationship at all to ‘10’ very good relationship. The ratings were averaged to obtain a mean score for the quality of the parental relationship. Higher scores indicated a better quality of the relationship with parents.

#### Daily Activity

Adolescents were asked whether they had a daily activity, (e.g., engaged in school or a job). A score of ‘0’ indicated that the adolescents did not have a daily activity (negative outcome) and a score of ‘1’ meant the adolescents was currently involved in school or work (positive outcome).

#### Social Network Other than Parents

Adolescents were asked how frequently they had contact with other family members other than their parents; for example, partner, best friend, other friends, and club or society (e.g., sports club). Answers were given on a 6-point scale ranging from ‘1’ never to ‘6’ daily. Scores were averaged to obtain a total score for social network. A higher score meant the adolescent had more frequent contact with their social network.

#### The Use of Soft Drugs

Adolescents were asked how often they used soft drugs in the last 6 months from ‘1’ monthly or less/none to ‘2’ weekly to ‘3’ daily. The higher the score, the more soft drugs were used.

#### Alcohol Use

Adolescents were asked about their alcohol consumption during the last 6 months. They answered on a scale ranging from ‘1’ monthly or less/not to ‘2’ weekly to ‘3’ daily. The higher the score on alcohol use, the more alcohol was used.

#### Internalizing Problems

Internalizing problems (anxiety or depression) were measured using the 16-item subscale on anxiety and depression from the Youth Self Report, Achenbach 1991; Achenbach and Rescorla 2001; Verhulst et al. 1997). Examples include ‘I have the feeling that I have to be perfect,’ ‘I feel worthless,’ and ‘I am unhappy, sad, or depressed.’ All items were rated on a 3-point scale, ‘1’ not at all to ‘3’ often. Cronbach’s alpha of this subscale was .84. A mean score was calculated and transferred into *t*-scores. Higher *t*-scores indicates more problem behaviors (i.e., more feelings of depression/anxiety). Because the *t*-scores were highly skewed, we transformed the scores into five categories: <51 (=0), 51–60 (=1), 61–70 (=2), 71–80 (=3) and >80 (=4).

#### Self-Reported Police Contacts

All adolescents were asked whether they had police contact since they were discharged. A score of ‘0’ meant the adolescent had had no police contact and a score of ‘1’ meant the adolescent had police contact in the last 6 months.

#### Official Police Contacts

Based on the national police systems, adolescents were traced to determine whether they had official police contact in the past 6 months and the frequency of police contact was registered. A higher score indicated a higher frequency of official police contact. For our chosen method of analysis we recoded the number of contacts as follows (between brackets the new code): 0 (0), 1 (1), 2 (2), 3 (3), 4 or 5 (4), 6 or 7 (5) and >7 (6).

### Procedure

The current study was part of a longitudinal study examining a new compulsory residential treatment program for adolescents with severe behavior problems. Six institutions participated, including thirty treatment groups. Of these treatment groups, thirteen groups consisted only of boys, nine groups consisted only of girls, and eight groups were mixed-sex treatment groups. Concerning the placement of adolescents in single-sex or mixed-sex treatment groups, the institutions made this determination. This is especially important for girls due to their vulnerability. Vulnerable girls (e.g., victims of prostitution or negative, traumatic sexual experiences), are typically placed in girls-only treatment groups. Next to the vulnerability of girls, the decision to place adolescents in single-sex or mixed-sex groups is based on problem behavior, age, and group composition. Two institutions offered the new treatment program to boys only. Regarding the remaining four institutions with both boys and girls, one consisted of only single-sex treatment groups, one of only mixed-sex treatment groups, and two of both single-sex and mixed-sex treatment groups. All adolescents entering the institutions between May 2007 and December 2009 participated in the study, *N* = 514. Because the sample consisted of underage adolescents with severe behavior problems, the study was reviewed and approved by the relevant medical ethics commission. Moreover, parental and adolescent consent was obtained allowing us to use their data for scientific purposes.

At time of admittance, the treatment files of these adolescents were analyzed to obtain insight into background characteristics regarding the period before admittance. During treatment the treatment improvement was measured. Using adolescents’ perceptions, Cohen’s *d* of .22 for internalizing problems and .42 for externalizing problems were found. For delinquency and drugs use the effect sizes were .30 and .29 respectively. For binge drinking a negative effect was found with a Cohen’s *d* of .41. At time of discharge, the institutions were asked to inform us of the date the adolescent left the institution and their discharge status. The reasons for discharge included regular (i.e., treatment goals were accomplished, treatment was finished in consultation with all involved parties) and non-regular discharge (i.e., the client ran away, youth care services ended treatment due to the clients’ misbehavior, treatment was ended because of the adolescent’s age (18+) or because judicial approval had expired). Six months after discharge, adolescents were approached by letter in which the aim of the study was briefly explained. Parents received a similar letter to ensure they were informed of the study as well. One week following the letter, adolescents were contacted by phone and asked for their willingness to participate. If adolescents could not be reached, a second letter was sent with a return letter and envelope. If interested, adolescents could provide their phone number and return the letter, after which contact was made. When adolescents could not be reached, contact was made with the adolescent’s guardian who was asked to inform the adolescent about the study and help us contact the adolescent. Confidentiality of the data was fully assured beforehand and adolescents received 25 euro for their participation.

A total of 420 adolescents (82%) were eligible to participate in the follow-up study. The other 94 adolescents had entered institutions before the beginning of our study, (i.e., before May 2007) and were not able to participate. Of the 420 eligible adolescents, 301 adolescents participated (59%). Of the other 119 participants, 11% did not want to participate, 12% could not be reached at the time of follow-up, and for 18% the researchers were not informed in time by the institutions about discharge.

Attrition analyses, using *t*-tests and χ²-tests, showed that the participating adolescents (*N* = 301) did not differ in the number of individual (*t*(514) = 2.58, *p* = .92), family (*t*(514) = .43, *p* = .15), and environmental (*t*(514) = .84, *p* = .44) risk factors at the time of entrance compared to the adolescents who did not participate (*N* = 213). Participating and non-participating adolescents were also equally distributed over treatment groups, (i.e., boys-only, girls-only, sex-mixed) *χ²*(514) = 4.45, *p* = .11. However, the two groups did differ in the duration of treatment. The participating adolescents had a significantly longer duration of treatment (*M* = 10.74 months, SD = 6.45) compared to non-participating adolescents (*M* = 9.57 months, SD = 6.15), *t*(514) = −1.97, *p* = .05. As a result, non-participating adolescents were more often non-regularly discharged (37%) than were participating adolescents (20%), *χ²*(514) = 17.57, *p* = .00.

## Results

First, descriptive statistics and correlations between all study variables were presented. Second, to examine the effects of the number of risk factors and treatment characteristics on post-treatment functioning for a sample of 301 adolescents admitted to compulsory residential care we used path analyses with age and ethnicity as control (independent) variables and the number of risk factors and treatment characteristics as predictors. Sex was not included as a control variable because one of the predictors was the sex composition of the treatment groups (girls-only, boys-only and mixed). Further, including all post-treatment indicators as dependent variables in one analysis would reduce the power drastically with a large number of parameters to be estimated. For this reason, we decided to estimate ten path models for each post-treatment indicator separately. The software package Mplus version 5.1 (Muthén and Muthén 2007) was used. To optimize the data, we used the full information estimator. Because the model variables were a mix of binary, ordered categorical, and interval variables, parameters were estimated using the Weighted Least Square with a Mean and Variance adjusted Chi-square test statistic estimator. Regression weights were expressed in probit coefficients and indicated the change in the *z*-score (or probit index) for a one unit change in the predictor. For this analysis, the adolescents were living in thirty groups, which meant the data could be dependent of this multilevel structure. To correct for non-independence (complexity) of the data because adolescents were nested within institutions, the COMPLEX procedure in Mplus was used to obtain unbiased estimates of the standard errors of the parameter estimates.

The composition of treatment groups (a structural treatment characteristic) consisted of three categories: girls-only treatment groups (g), boys-only treatment groups (b), and mixed-sex (m) treatment groups. Effects of the three groups were examined using unweighted effects coding (Cohen et al. 2003). Two codes represented the three groups g, b, m; one coded 1, 0, −1 and the second 0, 1, −1. Regression weights represented the deviation of the outcome variable for each separate group from the grand mean. However, only the effects of g and b were visible in the output. To determine the effect of m, a second analysis was conducted with a different coding system (−1, 1, 0 and −1, 0, 1) (Cohen et al. 2003). If effects were significant, post-hoc tests were applied. Dummy variables were created to compare g (1) with b (0), g (1) with m (0), and b (1) with m (0). Regression weights of the dummy variable were significant if the significance level was lower than .017 (known as Bonferroni correction with α = .05 divided by the number (3) of groups).

### Initial Analyses

#### Descriptive Statistics

The results showed that boys and girls differed in the number of individual and environmental risk factors. Girls were characterized by a significantly higher number of individual (*t*(1, 504) = −4.95, *p* = .00) and environmental risk factors (*t*(1, 504) = −6.18, *p* = .00) than were boys. On the family level, no differences were found between boys and girls. Regarding treatment characteristics, no differences were present between boys (*M* = 10.24 months, SD = 6.89) and girls (*M* = 10.39 months, SD = 5.68) the duration of treatment. Additionally, no differences were found between boys and girls for discharge status (70 and 77%, respectively, were regularly discharged). While the boys-only group consisted exclusively of boys and the girls-only group only of girls, the mixed group consisted of 48.6% boys and 51.4% girls. Table 1 shows the overall and sex-specific means and standard deviations for the ten post-treatment indicators. The results showed that significantly more girls than boys had a structured daily activity. Girls also used soft drugs less often and had fewer self-reported and official police contacts. However, boys experienced significantly fewer depressive symptoms and feelings of anxiety than did girls. Further, no differences were found between boys and girls for living situation, intensity and quality of contact with parents, social network, and alcohol consumption.

**Table 1:** Means and standard deviations of the post-treatment indicators for boys (*n* = 174) and girls (*n* = 127)

	Total		Boys		Girls		χ²/*z* ^a^/*F*
	*M*	SD	*M*	SD	*M*	SD	
Living situation							χ² = 2.38
Individual	19%		16%		24%		
Family	56%		59%		53%		
Residential/judicial	25%		25%		24%		
Intensity contact parents	3.46	.86	3.43	.90	3.49	.82	*z* = −.20
Quality relationship parents	7.67	1.58	7.71	1.60	7.58	1.57	*F* = .37
Daily activity							χ² = 5.72**
Having a daily activity	76%		70%		82%		
Not having a daily activity	24%		30%		18%		
Social network	3.54	1.08	3.53	1.12	3.56	1.03	*F* = .07
Use of soft drugs	1.38	.70	1.49	.75	1.24	.58	*z* = 3.36***
Alcohol consumption	2.03	.87	2.09	.86	1.94	.86	*z* = 1.46
Anxious/depressed	.61	.92	.44	.76	.83	1.08	*z* = −3.30***
Self-reported offending							
No offending	64%		57%		73%		
Offending	36%		43%		27%		
Official offending^b^	.54	1.15	.77	1.37	.21	.60	*z* = 5.39***
No offending	72%		64%		84%		
Offending	28%		36%		16%		

^a^This is the *z*-statistic of the Mann–Whitney test, ^b ^To be able to compare official offending with self-reported offending, we dichotomized official offending and included the percentages of adolescents who had police contact and adolescents who did not have police contacts after discharge

*p < .05; ** *p* < .01; *** *p* < .001

#### Correlations Between all Study Variables

Table 2 shows the correlations between the study variables. Because the study variables were a mix of interval, ordered categorical (ordinal variables), and binary variables, the correlations in Table 2 include polychoric (ordinal × ordinal and binary × ordinal variables), tetrachoric (binary × binary variables), biserial (interval × binary variables), polyserial (interval × ordinal variables), and Pearson (interval × interval variables) correlations. Additionally, because the full information estimator was used, correlations in this matrix were based on varying numbers of respondents. This explains why some of the lower correlations are significant and some of the higher correlations are sometimes nonsignificant.

**Table 2:** Correlations between all study variables

	1	2	3	4	5	6	7	8	9
1. Gender									
2. Age	.02								
3. Ethnicity	.17*	.08							
4. Individual RF	.27***	.17***	.16**						
5. Family RF	.02	−.16***	−.23***	.04					
6. Environmental RF	.33***	.10*	−.00	.31***	.04				
7. Duration of treatment	.02	−.16***	.08	−.04	−.02	.00			
8. Discharge status	−.15	.07	−.20*	−.05	.03	−.08	−.31***		
9. Living situation I	.18	.25**	.06	−.11	.16	−.09	.24**	−.27*	
10. Living situation II-.08	−.08	.23**	−.04	.01	−.21*	.09	.04	−.01	−.90***
11. Intensity family contact	.03	.06	.08	.01	−.11	.05	.02	−.07	−.43***
12. Quality family contact	−.05	−.07	−.07	.04	.00	.05	.00	−.05	−.19*
13. Daily activity	.21*	−.17*	.02	.02	−.03	.10	.05	−.07	.01
14. Social network	.02	.16**	.03	−.12*	−.12	.02	.10*	−.11	.11
15. The use of soft drugs	−.31***	.24**	.08	−.03	.12	−.10	−.00	.12	.12
16. Alcohol use	−.12	.16*	.35***	.08	−.12	−.06	.13*	−.26**	.14
17. Anxious/depressed	.30***	−.02	−.09	.04	.06	.02	−.01	.02	.12
18. Self-reported offending	−.26**	−.03	−.04	.06	.13	−.10	.10	−.08	.05
19. Official offending	−.42***	.04	.02	.03	−.00	.05	−.05	.10	−.09

	10	11	12	13	14	15	16	17	18
1. Gender									
2. Age									
3. Ethnicity									
4. Individual RF									
5. Family RF									
6. Environmental RF									
7. Duration of treatment									
8. Discharge status									
9. Living situation I									
10. Living situation II-.08									
11. Intensity family contact	.64***								
12. Quality family contact	.08	.33***							
13. Daily activity	−.09	.06	.18*						
14. Social network	.24***	.21**	.14**	.11					
15. The use of soft drugs	.07	−.08	−.21**	−.17	−.05				
16. Alcohol use	.25**	.12	−.06	.10	.34***	.25**			
17. Anxious/depressed	−.42***	−.24**	−.30***	−.17	−.18**	.12	−.15		
18. Self-reported offending	−.01	.01	−.16*	−.24*	−.09	.27**	.04	.00	
19. Official offending	.02	.05	.02	−.12	−.02	.10	−.04	−.15	.69***

RF = risk factors; Living Situation I = individual versus family and residential/judicial; Living Situation II = family versus individual and residential/judicial

* *p* < .05; ** *p* < .01; *** *p* < .001

### Path Analyses

To examine the association between structural treatment characteristics and post-treatment functioning, path analyses were applied. The number of risk factors was also included in this model. The fit of the path models was good. Each of the ten path models showed almost identical fit values with Chi-square = 21.80, *df* = 18 and *p* = .241 for all models. The RMSEA-values were .021 and CFI-values ranged between .948 and .983. Regarding risk factors, only the number of risk factors within the family was related to indicators of post-treatment functioning. The higher the number of family risk factors, the more likely an adolescent was to live on his or her own and the more likely he or she was to have had self-reported police contacts within 6 months after discharge. The number of individual and environmental risk factors was not predictive of post-treatment functioning when structural treatment characteristics were included in the model (see Table 3).

**Table 3:** Regression analyses regarding the association between structural treatment characteristics and post-treatment functioning

	Living situation I	Living situation II	Intensity family contact	Quality family contact	Daily activity	Social net-work	The use of soft drugs	Alcohol use	Anxious/depressed	Self-reported offending	Official offending
	*B*	*B*	*B*	*B*	*B*	*B*	*B*	*B*	*B*	*B*	*B*
Age	.26**	.17*	.06	−.11	−.16***	.16	.23***	.16**	−.01	.01	−.00
Ethnicity	.12	−.12	.05	−.23	−.06	.04	.32	.49***	−.19	−.03	.14
Individual RF	−.04	.01	−.02	.00	−.01	−.08	.00	.05	.02	.06	.01
Family RF	.10	−.06*	−.03	−.02	−.03	−.02	.09	−.02	.01	.05*	.01
Environmental RF	−.19	.22	.19	.19	.16	.08	−.20	−.15	−.03	−.23	.08
Duration of treatment	−.04**	.02	−.09	−.00	.00	.02	.01	.01	.00	.02	−.01
Discharge status	−.15	−.03	−.16*	−.01	−.07	−.10	.14	−.27**	.01	−.06	.07
Treatment groups											
Girls-only	.18**	−.12	−.16	−.29	.06	.04	−.24*	−.15*	.25*	−.05	−.18
Boy-only	−.15	.08	.02	.05	−.18	.07	.38*	.17	−.31*	.27**	.40***
Mixed-sex	−.02	.04	.13	.23	.14	−.12	−.16	−.04	.08	−.24	−.22

RF = risk factors; Living Situation I = individual versus family and residential/judicial; Living Situation II = family versus individual and residential/judicial

* *p* < .05; ** *p* < .01; *** *p* < .001

Concerning structural treatment characteristics, duration of treatment was found to be related to the living situation after discharge, in that adolescents who stayed for a longer period were more likely to live in a residential judicial setting or family situation 6 months after discharge. For discharge status, adolescents with a regular discharge showed a higher intensity of family contact and higher alcohol consumption 6 months later. Concerning the composition of treatment groups, adolescents in the girls-only groups were more likely to live independently, showed less alcohol and drug use, and were more likely to experience feelings of anxiety or depression compared to the overall means on these variables. Adolescents in the boys-only groups used more soft drugs, were less anxious or depressed, and scored higher on self-reported and official offending compared to the overall means on these variables. The mixed groups did not show significant effects for any of the post-treatment indicators (see Table 3).

Based on the above analysis, it was only possible to determine whether the groups differed from the overall mean; therefore, the next step was to examine whether groups differed from each other. For significant results (living situation, alcohol and drug use, feelings of depression or anxiety, self-reported and official offending), we applied *post hoc* testing to detect significant differences between girls-only (1) and boys-only groups (0), girls-only (1) and mixed groups (0), and boys-only (1) and mixed groups (0). Only significant findings will be presented below. For living situation, the girls-only groups more often lived on their own than did the boys-only groups (*B* = .26, *p* = .021) and the mixed groups (*B* = .20, *p* = .023). After a Bonferroni correction, these results were not significant. Use of soft drugs and alcohol were significantly higher for the boys-only groups than for the girls-only groups (*B* = −.41, *p* = .003, for soft drugs; *B* = −.19, *p* = .002, for alcohol use). Feelings of anxiety or depression were significantly higher for the girls-only groups compared to the boys-only groups (*B* = .43, *p* = .000) and significantly higher for the mixed-groups compared to the boys-only groups (*B* = −.27, *p* = .006). Self-reported offending revealed a significant effect for the boys-only groups compared to the mixed groups (*B* = .29, *p* = .012), indicating that the boys-only groups self-reported more offending. For official offending, the boys-only groups showed more official police contacts after treatment than did the girls-only groups (*B* = −.41, *p* = .000) and the mixed groups (*B* = .38, *p* = .000).

For the variable internalizing problems (anxiety/depression), self-reported offending, and official offending findings suggest significant effects between single-sex and mixed-sex treatment groups. The question then arises whether, besides sex effects, a group composition effect existed. We compared the girls-only (1) with the girls from the mixed groups (0) and the boys-only (1) with the boys from the mixed groups (0). For internalizing problems and self-reported offending, no significant differences between the two groups were found. This means that the effects of these two variables are probably sex effects and not group composition effects. For the variable official offending, results revealed that girls in the mixed groups showed significantly fewer police contacts than did the girls in the girls-only groups (*B* = .64, *p* = .000). No significant differences between the boys in the single-sex and boys in the mixed-sex groups (*B* = .09, *p* = .465) were found. These results indicate that a positive group composition effect existed: 4% of girls in the mixed groups had official police contact compared to 21% of girls in the girls-only groups.

## Discussion

The primary aim of the present study was to examine the effects of structural treatment characteristics (duration of treatment, discharge status, and group composition) on post-treatment functioning 6 months after discharge. Concerning our hypotheses, we did expect that a longer duration of treatment and regular discharge would be related to more positive outcomes. Our hypotheses were not confirmed, duration of treatment and discharge status were hardly related to treatment outcomes. Regarding group composition, we expected that girls in girls-only treatment groups would do better after treatment. Also group composition was hardly related to treatment outcomes, except for criminal behavior. Girls in girls-only treatment groups were more likely to have police contacts after treatment, which is in contrast with our hypothesis. Overall, structural treatment characteristics were hardly related to treatment outcomes.

Findings of Casey et al. (2010) suggest that adolescents have some concerns about the transition from a treatment institution to society; primarily whether contact with their parents will be positive. The results of our study revealed an overall positive contact with parents 6 months after discharge. Nevertheless, adolescents who were confronted with a higher number of family risk factors were found to live independently more often. Furthermore, when adolescents reported lower quality of contact with parents, they more often lived on their own and had less frequent contact with their parents. To conclude, the adolescents in our study were, in general, positive about the contact with their parents; however, this depended on the number of family risk factors at time of entrance.

A second concern of adolescents leaving treatment is the influence of deviant friends (Casey et al. 2010). We found that adolescents who committed crimes after treatment were more often male, more likely to report lower quality of contact with their parents, had less contact with their parents, and had more frequent contact with their social network other than parents. This might indicate that adolescents, especially those who do not have a good relationship with parents in terms of frequency and quality of contact, are more likely to focus on their social network after discharge, which possibly includes a deviant network. Based on previous studies, it is known that the parenting environment is related to involvement in deviant peer groups (Dishion et al. 2004, 1996). Low parental monitoring is related to a higher likelihood of becoming involved with deviant friends (Dishion et al. 2004). Additionally, Ary et al. (1999) found that a high level of family conflict leads to less parental involvement and inadequate parental monitoring, which increases the involvement in deviant peer groups. Overall, this suggests that it is important to intensively intervene on family risk factors within the new treatment program. Despite the availability of various family interventions (e.g., Functional Family Therapy, Multisystemic Therapy) within the new treatment program, future studies should explore specific family risk factors that exert a large impact and are related to post-treatment functioning, in order to better develop appropriate interventions on the family level.

Adolescents appeared to do relatively well 6 months after discharge on several post-treatment indicators. Examining the influence of treatment characteristics on this post-treatment functioning indicated that the duration of treatment was not related to how well the adolescents did after treatment, except for the living situation. Adolescents in treatment for shorter durations were more likely to live on their own 6 months after discharge. Findings also revealed that living independently was related to higher levels of family risk factors. It might be that adolescents who have family risk factors, rather than individual risk factors, may be less in need of intensive treatment and may be discharged earlier than adolescents who are characterized by high levels of individual risk factors. Our findings contrast with those of Trout et al. (2010), who found that adolescents living on their own after discharge underwent a longer duration of treatment. The fact that adolescents in treatment for shorter durations were more likely to live on their own in the Netherlands, as opposed to the U.S., might be due to the fact that the Dutch welfare system provides financial support and can offer housing to adolescents who cannot return to their parents. These possibilities offered by the Dutch welfare system may shorten the treatment period for adolescents in the Netherlands.

From recent studies it is known that girls enter residential care with more troubled behaviour (Connor et al. 2004; Doerfler et al. 2009; Nijhof 2011). Also some sex differences in the adolescents functioning after treatment emerged in our study: girls more often had a structured daily activity, used less soft drug, and had fewer police contacts than boys, but higher levels of depression and anxiety. Fields and Abrams (2010) showed some similar findings. They found differences between boys and girls in their needs related to re-entry into society. They found that girls showed higher levels of anxiety about their returning home, showed higher levels of mental health problems. During treatment boys and girls can be placed in boys-only, girls-only and sex-mixed treatment groups. The effect of group composition on treatment outcomes is largely understudied in the field of residential youth care. Studies including adult females have suggested that sex differences are not adequately taken into account in mixed-sex treatment groups and treatment needs are better met in single-sex treatment programs (Kauffman et al. 2010). On the other hand, one developmental task during adolescence is to instigate and develop (sexual) romantic relationships (Verhofstadt-Denève et al. 1995), which might be better met in mixed-sex treatment groups. One major finding of our study is that few differences were found in post-treatment outcomes between the treatment groups. Although differences were found for anxiety and depression and self-reported offending, these differences could be explained by sex rather than group composition effects. One important difference between treatment groups, not due to sex effects, did emerge: girls in mixed-sex treatment groups had significantly fewer official police contacts than did girls in single-sex groups. Examining this compelling finding more closely reveals that, compared to girls in mixed-sex groups, significantly more girls in single-sex groups started to commit crimes after treatment (‘starters’). It is possible that deviancy training, in which peers reinforce one another’s deviant behaviors (Dishion et al. 1996, 1997, 2001) is stronger in single-sex treatment groups than in mixed-sex groups. It is known that deviancy training within girls-only groups occurs in the new residential treatment program (De Haan et al. 2010); however, whether it also takes place in mixed-sex groups remains to be answered (T. J. Dishion, personal communication, January 25, 2011). It should be noted that there are indications that the institutions under study placed girls in single-sex or mixed-sex treatment groups based on these girls’ problem behaviors and vulnerability. Girls who are more traumatized, that is, girls who have a history of a sexual abuse or who are victims of prostitution, are more likely to be placed in girls-only treatment groups rather than in mixed-sex groups. This difference in placement criteria for girls might be related to our finding, in that the specific problem behaviors are related to a higher vulnerability for peer contagion. As is suggested in the study of Fields and Abrams (2010), we agree in that sex-specific needs must be better met during and after treatment both in the practical field as well as in scientific research. For example, more research on deviancy training within treatment groups is needed and should include both single-sex and mixed-sex treatment groups to compare boys and girls in the different types of treatment groups.

### Limitations of the Study

Some limitations need to be mentioned. First, a well-known and common limitation in evaluating a treatment program, like ours, is that the study includes a single sample design (e.g., Kazdin 1993; Little et al. 2005). Because no control group was included, no conclusions can be drawn about causality between treatment characteristics and outcomes. Therefore, it still remains unknown whether the adolescents would show similar results if they did not receive residential treatment. A second limitation of this study was that some adolescents in the treatment program did not participate. While the adolescents who participated did not differ on the number of risk factors before admittance from those who did not participate, they did differ in duration and discharge status. Adolescents who did not participate in the current study stayed in treatment for shorter periods and were more often non-regularly discharged. According to Sunseri (2001) some factors associated with non-completion of the program include parental illnesses, legal status of the child, history of residential care, a combination of problem behaviors, and a diagnosis of post traumatic stress disorder. Except for parental illness, which was one of the family risk factors in the current study, we did not include these factors in the risk model. It is possible that adolescents who did not participated in the present study differed on the factors mentioned by Sunseri. Third, according to Hair (2005), there are three distinctive factors positively related to the maintenance of treatment improvement: involvement of parents during treatment, stability of the post-treatment environment, and after care for both the adolescent and the family. Although parents were involved in the new treatment program and contact with parents remained positive 6 months after treatment, stability of the post-treatment environment and aftercare were not considered. A fourth limitation concerns the placement of adolescents. As mentioned earlier the placement of adolescent boys and girls is based on the problem behavior at time of entry, the age, the vulnerability en group composition. In prior studies it was found that group care workers behavior and group climate are associated with the adolescents’ problem behavior and treatment outcomes (Marsh et al. 2010; Van Dam et al. 2011; Van der Helm et al. 2009). Although the aim of the present study was not to investigate group climate or group care worker behavior, this might be possible confounding factors. Also the specific interventions given to an individual were not included because this information was known of only a small sample. In future studies these possible confounding factors should be included or at least controlled for. Fifth, the present study also did not include data on behavioral changes during treatment, while this might have influenced the outcomes. Previous reviews conclude that residential treatment improves the adolescents’ functioning (e.g., Bettman and Jasperson 2009; Frensch and Cameron 2002; Knorth et al. 2008). A previous study examining the same sample found that adolescents do show improvement during treatment concerning internalizing problems with effect sizes of .22 according to the adolescents’ perception and .59 according to the parents’ perception. For externalizing problems, these effect sizes were .42 and .78 respectively (Nijhof et al. 2011). Knorth et al. (2008) found similar effect sizes ranging between .45 and .60. Future studies should include treatment improvement when examining the adolescents’ functioning after treatment by using the reliable change index. The reliable change index provides information about the individuals’ change between the start and the end of treatment (Jacobson and Truax 1991; Hafkenscheid et al. 1998; Veerman 2007).

### Conclusion

To conclude, our study showed that a few treatment characteristics are predictive for post-treatment functioning. The associations found suggest an intensive focus on the family level, especially for families demonstrating a high level of family risk factors. Moreover, our study adds to our understanding of the impact of placing boys and girls in single-sex versus mixed-sex treatment groups on individual development and functioning. Further research is needed to better understand the mechanism and various possible impact of peer contagion in boys-only, girls-only and mixed-sex treatment groups.
